# 4,4,4-Trifluoro-3-hy­droxy-3-(trifluoro­meth­yl)butanoic acid

**DOI:** 10.1107/S1600536811004764

**Published:** 2011-02-12

**Authors:** Richard Betz, Thomas Gerber, Henk Schalekamp

**Affiliations:** a Nelson Mandela Metropolitan University, Summerstrand Campus, Department of Chemistry, University Way, Summerstrand, PO Box 77000, Port Elizabeth 6031, South Africa

## Abstract

The asymmetric unit of the title compound, C_5_H_4_F_6_O_3_, a polyfluorinated derivative of β-hy­droxy­butyric acid, comprises two mol­ecules. Intra­molecular O—H⋯O hydrogen bonds occur. In the crystal, inter­molecular O—H⋯O hydrogen bonds give rise to the formation of carb­oxy­lic acid dimers. Along with these hydrogen bonds, C—H⋯O contacts connect the mol­ecules into infinite strands along the *a* axis.

## Related literature

For the crystal structure of (*S*)-3-amino-4,4,4-trifluoro­butane­carb­oxy­lic acid, see: Soloshonok *et al.* (1993 ▶). For the graph-set analysis of hydrogen bonds, see: Etter *et al.* (1990 ▶); Bernstein *et al.* (1995 ▶).
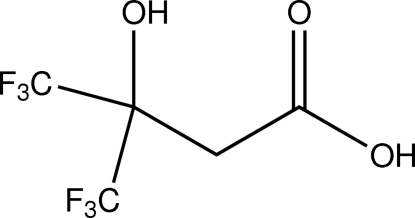


## Experimental

### 

#### Crystal data

C_5_H_4_F_6_O_3_
*M* *_r_* = 226.08
Monoclinic, 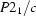

*a* = 5.5031 (2) Å
*b* = 20.5490 (8) Å
*c* = 14.0342 (6) Å
β = 98.4543 (14)°
*V* = 1569.79 (11) Å^3^
*Z* = 8
Mo *K*α radiation
μ = 0.24 mm^−1^
*T* = 200 K
0.59 × 0.45 × 0.33 mm

#### Data collection

Bruker APEXII CCD diffractometer
25936 measured reflections
3897 independent reflections
3469 reflections with *I* > 2σ(*I*)
*R*_int_ = 0.014

#### Refinement

*R*[*F* ^2^ > 2σ(*F* ^2^)] = 0.038
*wR*(*F* ^2^) = 0.100
*S* = 1.04
3897 reflections
257 parameters
H-atom parameters constrained
Δρ_max_ = 0.42 e Å^−3^
Δρ_min_ = −0.38 e Å^−3^

### 

Data collection: *APEX2* (Bruker, 2010 ▶); cell refinement: *SAINT* (Bruker, 2010 ▶); data reduction: *SAINT*; program(s) used to solve structure: *SHELXS97* (Sheldrick, 2008 ▶); program(s) used to refine structure: *SHELXL97* (Sheldrick, 2008 ▶); molecular graphics: *ORTEP-3 for Windows* (Farrugia, 1997 ▶) and *Mercury* (Macrae *et al.*, 2006 ▶); software used to prepare material for publication: *SHELXL97* and *PLATON* (Spek, 2009 ▶).

## Supplementary Material

Crystal structure: contains datablocks I, global. DOI: 10.1107/S1600536811004764/gk2344sup1.cif

Structure factors: contains datablocks I. DOI: 10.1107/S1600536811004764/gk2344Isup2.hkl

Additional supplementary materials:  crystallographic information; 3D view; checkCIF report

## Figures and Tables

**Table 1:** Hydrogen-bond geometry (Å, °)

*D*—H⋯*A*	*D*—H	H⋯*A*	*D*⋯*A*	*D*—H⋯*A*
O1—H1⋯O5	0.84	1.83	2.6653 (14)	178
O3—H3⋯O2	0.84	2.01	2.7296 (14)	144
O3—H3⋯O1^i^	0.84	2.45	2.9561 (15)	120
O4—H4⋯O2	0.84	1.82	2.6635 (13)	178
O6—H6⋯O5	0.84	2.03	2.7502 (13)	144
O6—H6⋯O4^ii^	0.84	2.41	2.9276 (13)	121
C2—H2*A*⋯O3^ii^	0.99	2.36	3.2773 (16)	153
C7—H7*A*⋯O6^i^	0.99	2.32	3.2340 (14)	153

Symmetry codes: (i) 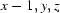; (ii) 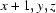.

**Fig. 1.**
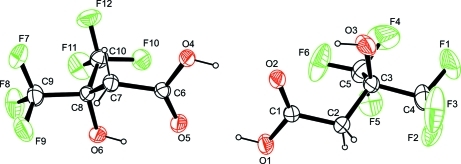
The asymmetric unit of the title compound with anisotropic displacement ellipsoids drawn at the 50% probability level.

**Fig. 2.**
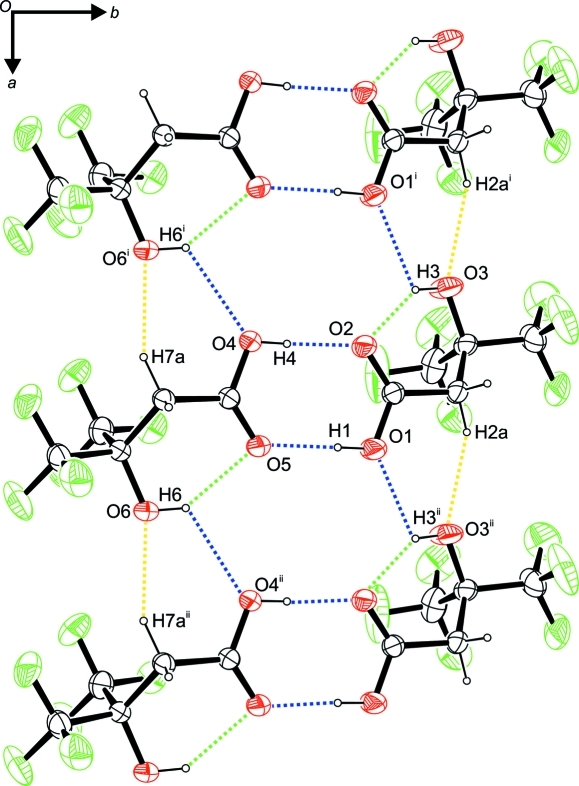
Intermolecular C–H···O contacts and hydrogen bonds in the title compound, viewed along [0 0 - 1]. Intramolecular hydrogen bonds are indicated with green, intermolecular hydrogen bonds with blue dotted lines. The C–H···O contacts are illustrated with yellow dotted lines. Symmetry operators: i -1 + x, y, z; ii = 1 + x, y, z.

## supplementary crystallographic information

### Comment

Chelate ligands have found widespread use in coordination chemistry due to the increased stability of coordination compounds they can form in comparison to monodentate ligands. Most work in this field has been done with chelate ligands capable of forming five-, six- and seven-membered chelate rings. The coordination behaviour of such ligands with respect to reaction products formed (*e.g.* the coordination number of the central atom) is a function of electronic as well as steric factors. In a larger study aimed at elucidating the coordination chemistry of multiply-fluorinated carboxylic acid derivatives, the structure of the title compound was determined to enable comparisons with reaction products obtained.

The title compound is a symmetric, polyhalogenated derivative of β-hydroxypropanecarboxylic acid which bears two trifluoromethyl-groups at the alcoholic carbon atom. The asymmetric unit (Fig. 1) comprises two molecules of the title compound.

In the crystal structure, intra- as well as intermolecular hydrogen bonds are present. While the intramolecular hydrogen bonds are formed between the alcoholic hydroxyl group and the carbonylic O-atom of the carboxylic group, intermolecular hydrogen bonds can be observed between the carboxylic acid groups' OH-groups and carbonylic O-atoms. The latter interaction connects both molecules of the asymmetric unit to dimers. In terms of graph-set analysis, the descriptor for the intramolecular hydrogen bonds is *S*(6)*S*(6) on the unitary level while the intermolecular hydrogen bonds necessitate a *R*^2^_2_(8) descriptor on the binary level. For the intramolecular hydrogen bond, a bifurcation could be discussed applying the O-atom of another hydroxyl group as acceptor. This would render it a mixed intra-intermolecular hydrogen bond, however, the D–H···A angle of only around 120° for the intermolecular hydrogen bond is comparatively small.

Apart from these hydrogen bonds, C–H···O contacts are present in the crystal structure whose ranges fall more than 0.2 Å below the sum of van-der-Waals radii of the respective atoms. These contacts can be observed between one of the H-atoms of the methylene group and the O-atom of a neighbouring hydroxyl group. Like the possible, bifurcated hydrogen bond mentioned above, these C–H···O contacts connect the molecules to infinite strands along the crystallographic *a* axis (Fig. 3). The descriptor for the C–H···O contacts on the unitary level is *C*^1^_1_(4)*C*^1^_1_(4).

### Experimental

The structural analysis was done on a single-crystal taken from a commercially obtained (Fluorochem) batch of the title compound.

### Refinement

Carbon-bound H-atoms were placed in calculated positions (C—H 0.99 Å) and were included in the refinement in the riding model approximation, with *U*(H) set to 1.2*U*_eq_(C). The H-atoms of the carboxylic acid group as well as of the hydroxyl groups were allowed to rotate with a fixed angle around the C—O bond to best fit the experimental electron density (HFIX 147 in the *SHELX* program suite (Sheldrick, 2008)), their *U*(H) invariably set to 1.5*U*_eq_(C)

### Crystal data

**Table:**

C_5_H_4_F_6_O_3_	*F*(000) = 896
*M**_r_* = 226.08	*D*_x_ = 1.913 Mg m^−^^3^
Monoclinic, *P*2_1_/*c*	Mo *K*α radiation, λ = 0.71073 Å
Hall symbol: -P 2ybc	Cell parameters from 9089 reflections
*a* = 5.5031 (2) Å	θ = 2.9–28.3°
*b* = 20.5490 (8) Å	µ = 0.24 mm^−^^1^
*c* = 14.0342 (6) Å	*T* = 200 K
β = 98.4543 (14)°	Rod, colourless
*V* = 1569.79 (11) Å^3^	0.59 × 0.45 × 0.33 mm
*Z* = 8	

### Data collection

**Table:**

Bruker APEXII CCD diffractometer	3469 reflections with *I* > 2σ(*I*)
Radiation source: fine-focus sealed tube	*R*_int_ = 0.014
graphite	θ_max_ = 28.3°, θ_min_ = 2.5°
φ and ω scans	*h* = −7→7
25936 measured reflections	*k* = −27→27
3897 independent reflections	*l* = −18→18

### Refinement

**Table:**

Refinement on *F*^2^	Primary atom site location: structure-invariant direct methods
Least-squares matrix: full	Secondary atom site location: difference Fourier map
*R*[*F*^2^ > 2σ(*F*^2^)] = 0.038	Hydrogen site location: inferred from neighbouring sites
*wR*(*F*^2^) = 0.100	H-atom parameters constrained
*S* = 1.04	*w* = 1/[σ^2^(*F*_o_^2^) + (0.0438*P*)^2^ + 0.8289*P*] where *P* = (*F*_o_^2^ + 2*F*_c_^2^)/3
3897 reflections	(Δ/σ)_max_ < 0.001
257 parameters	Δρ_max_ = 0.42 e Å^−^^3^
0 restraints	Δρ_min_ = −0.38 e Å^−^^3^

### Fractional atomic coordinates and isotropic or equivalent isotropic displacement parameters (Å^2^)

**Table:**

	*x*	*y*	*z*	*U*_iso_*/*U*_eq_	
O1	0.86328 (18)	0.71888 (5)	0.08430 (10)	0.0429 (3)	
H1	0.8645	0.6811	0.1073	0.064*	
O2	0.45964 (17)	0.70927 (5)	0.08404 (8)	0.0326 (2)	
O3	0.17625 (19)	0.80005 (7)	−0.02218 (11)	0.0520 (4)	
H3	0.2000	0.7657	0.0103	0.078*	
O4	0.47274 (16)	0.59040 (4)	0.16029 (7)	0.0285 (2)	
H4	0.4717	0.6280	0.1367	0.043*	
O5	0.87553 (17)	0.59872 (5)	0.15635 (8)	0.0337 (2)	
O6	1.13316 (16)	0.48391 (5)	0.18383 (8)	0.0316 (2)	
H6	1.1185	0.5241	0.1733	0.047*	
C1	0.6381 (2)	0.74043 (6)	0.06831 (9)	0.0261 (2)	
C2	0.6189 (2)	0.80980 (6)	0.03285 (10)	0.0281 (3)	
H2A	0.7714	0.8211	0.0069	0.034*	
H2B	0.6071	0.8389	0.0882	0.034*	
C3	0.3986 (2)	0.82251 (7)	−0.04523 (10)	0.0301 (3)	
C4	0.3661 (3)	0.89678 (8)	−0.05702 (13)	0.0431 (4)	
C5	0.4440 (4)	0.79138 (8)	−0.14087 (12)	0.0477 (4)	
C6	0.6980 (2)	0.56870 (6)	0.17642 (9)	0.0233 (2)	
C7	0.7220 (2)	0.50386 (6)	0.22712 (9)	0.0246 (2)	
H7A	0.5583	0.4829	0.2201	0.030*	
H7B	0.7761	0.5113	0.2967	0.030*	
C8	0.9032 (2)	0.45714 (6)	0.18926 (9)	0.0237 (2)	
C9	0.9528 (3)	0.40037 (7)	0.26098 (11)	0.0343 (3)	
C10	0.7933 (3)	0.43236 (7)	0.08834 (10)	0.0333 (3)	
F1	0.1814 (3)	0.91197 (7)	−0.12449 (13)	0.0885 (5)	
F2	0.5634 (2)	0.92505 (5)	−0.08100 (10)	0.0611 (3)	
F3	0.3264 (3)	0.92357 (6)	0.02449 (11)	0.0842 (5)	
F4	0.2561 (3)	0.79879 (8)	−0.21017 (9)	0.0852 (5)	
F5	0.6404 (2)	0.81581 (6)	−0.17319 (8)	0.0595 (3)	
F6	0.4835 (4)	0.72803 (6)	−0.12768 (9)	0.0885 (5)	
F7	0.74664 (19)	0.36955 (5)	0.27353 (8)	0.0483 (2)	
F8	1.1068 (2)	0.35676 (5)	0.23401 (9)	0.0582 (3)	
F9	1.05355 (19)	0.42287 (5)	0.34686 (7)	0.0486 (3)	
F10	0.75894 (18)	0.48279 (5)	0.02779 (6)	0.0430 (2)	
F11	0.9385 (2)	0.39072 (6)	0.05224 (8)	0.0601 (3)	
F12	0.57504 (19)	0.40413 (5)	0.08745 (7)	0.0503 (3)	

### Atomic displacement parameters (Å^2^)

**Table:**

	*U*^11^	*U*^22^	*U*^33^	*U*^12^	*U*^13^	*U*^23^
O1	0.0233 (5)	0.0342 (5)	0.0710 (8)	0.0020 (4)	0.0066 (5)	0.0233 (5)
O2	0.0239 (4)	0.0293 (5)	0.0446 (6)	0.0004 (4)	0.0053 (4)	0.0128 (4)
O3	0.0223 (5)	0.0557 (7)	0.0775 (9)	−0.0007 (5)	0.0055 (5)	0.0399 (7)
O4	0.0201 (4)	0.0232 (4)	0.0421 (5)	0.0019 (3)	0.0042 (4)	0.0057 (4)
O5	0.0215 (4)	0.0276 (5)	0.0525 (6)	0.0005 (4)	0.0068 (4)	0.0101 (4)
O6	0.0181 (4)	0.0299 (5)	0.0474 (6)	0.0013 (3)	0.0061 (4)	0.0051 (4)
C1	0.0243 (6)	0.0250 (6)	0.0284 (6)	−0.0002 (5)	0.0021 (5)	0.0039 (5)
C2	0.0260 (6)	0.0232 (6)	0.0343 (6)	−0.0023 (5)	0.0020 (5)	0.0039 (5)
C3	0.0252 (6)	0.0282 (6)	0.0368 (7)	−0.0012 (5)	0.0042 (5)	0.0117 (5)
C4	0.0451 (8)	0.0333 (7)	0.0536 (9)	0.0100 (6)	0.0162 (7)	0.0177 (7)
C5	0.0688 (11)	0.0382 (8)	0.0333 (8)	−0.0069 (8)	−0.0022 (7)	0.0056 (6)
C6	0.0215 (5)	0.0224 (5)	0.0258 (6)	0.0009 (4)	0.0026 (4)	−0.0009 (4)
C7	0.0223 (5)	0.0241 (6)	0.0284 (6)	0.0026 (4)	0.0069 (4)	0.0039 (5)
C8	0.0205 (5)	0.0223 (5)	0.0284 (6)	0.0016 (4)	0.0042 (4)	0.0024 (4)
C9	0.0363 (7)	0.0283 (6)	0.0384 (7)	0.0067 (5)	0.0058 (6)	0.0077 (5)
C10	0.0377 (7)	0.0301 (7)	0.0320 (7)	−0.0014 (5)	0.0050 (5)	−0.0032 (5)
F1	0.0713 (9)	0.0650 (8)	0.1198 (13)	0.0159 (7)	−0.0168 (8)	0.0530 (8)
F2	0.0698 (7)	0.0310 (5)	0.0890 (9)	−0.0065 (5)	0.0329 (6)	0.0162 (5)
F3	0.1413 (14)	0.0428 (6)	0.0828 (9)	0.0398 (7)	0.0640 (9)	0.0165 (6)
F4	0.0932 (10)	0.1107 (12)	0.0420 (6)	−0.0324 (9)	−0.0229 (6)	0.0152 (7)
F5	0.0693 (7)	0.0685 (7)	0.0459 (6)	0.0097 (6)	0.0255 (5)	0.0052 (5)
F6	0.1863 (17)	0.0330 (6)	0.0454 (6)	−0.0033 (8)	0.0140 (8)	−0.0057 (5)
F7	0.0554 (6)	0.0367 (5)	0.0539 (6)	−0.0085 (4)	0.0122 (5)	0.0157 (4)
F8	0.0696 (7)	0.0405 (5)	0.0674 (7)	0.0310 (5)	0.0197 (6)	0.0141 (5)
F9	0.0552 (6)	0.0490 (6)	0.0369 (5)	0.0086 (5)	−0.0084 (4)	0.0121 (4)
F10	0.0524 (5)	0.0473 (5)	0.0275 (4)	−0.0056 (4)	0.0000 (4)	0.0059 (4)
F11	0.0746 (8)	0.0577 (7)	0.0495 (6)	0.0186 (6)	0.0140 (5)	−0.0199 (5)
F12	0.0496 (6)	0.0501 (6)	0.0485 (6)	−0.0230 (5)	−0.0022 (4)	−0.0053 (4)

### Geometric parameters (Å, °)

**Table:**

O1—C1	1.3042 (16)	C7—C8	1.5347 (16)
O1—H1	0.8400	C7—H7A	0.9900
O2—C1	1.2192 (16)	C7—H7B	0.9900
O3—C3	1.3896 (16)	C8—C9	1.5387 (18)
O3—H3	0.8400	C8—C10	1.5425 (19)
O4—C6	1.3055 (15)	F1—C4	1.321 (2)
O4—H4	0.8400	F2—C4	1.3183 (19)
O5—C6	1.2228 (15)	F3—C4	1.316 (2)
O6—C8	1.3920 (14)	F4—C5	1.320 (2)
O6—H6	0.8400	F5—C5	1.330 (2)
C1—C2	1.5084 (17)	F6—C5	1.328 (2)
C2—C3	1.5325 (18)	F7—C9	1.3336 (18)
C2—H2A	0.9900	F8—C9	1.3259 (17)
C2—H2B	0.9900	F9—C9	1.3330 (19)
C3—C5	1.540 (2)	F10—C10	1.3359 (17)
C3—C4	1.543 (2)	F11—C10	1.3213 (17)
C7—C6	1.5073 (17)	F12—C10	1.3323 (17)
			
O1—C1—C2	113.34 (11)	C8—C7—H7A	108.8
O2—C1—O1	124.10 (12)	C8—C7—H7B	108.8
O2—C1—C2	122.48 (11)	C9—C8—C10	111.00 (11)
O3—C3—C2	114.09 (11)	F1—C4—C3	112.06 (16)
O3—C3—C4	105.15 (12)	F2—C4—C3	112.11 (13)
O3—C3—C5	109.04 (13)	F2—C4—F1	106.79 (14)
O4—C6—C7	113.50 (10)	F3—C4—C3	110.61 (13)
O5—C6—O4	123.94 (11)	F3—C4—F1	108.40 (16)
O5—C6—C7	122.50 (11)	F3—C4—F2	106.61 (16)
O6—C8—C7	114.46 (10)	F4—C5—C3	112.59 (17)
O6—C8—C9	105.08 (10)	F4—C5—F5	107.03 (14)
O6—C8—C10	108.49 (10)	F4—C5—F6	108.05 (16)
C1—O1—H1	109.5	F5—C5—C3	112.62 (14)
C1—C2—C3	113.99 (11)	F6—C5—C3	109.26 (13)
C1—C2—H2A	108.8	F6—C5—F5	107.04 (18)
C1—C2—H2B	108.8	F7—C9—C8	111.95 (11)
C2—C3—C4	108.21 (12)	F8—C9—C8	112.64 (12)
C2—C3—C5	109.85 (12)	F8—C9—F7	107.96 (13)
C3—O3—H3	109.5	F8—C9—F9	107.12 (12)
C3—C2—H2A	108.8	F9—C9—C8	109.80 (12)
C3—C2—H2B	108.8	F9—C9—F7	107.10 (12)
C5—C3—C4	110.40 (12)	F10—C10—C8	109.13 (11)
C6—O4—H4	109.5	F11—C10—C8	112.85 (12)
C6—C7—C8	113.94 (10)	F11—C10—F10	107.19 (12)
C6—C7—H7A	108.8	F11—C10—F12	108.03 (13)
C6—C7—H7B	108.8	F12—C10—C8	112.56 (11)
C7—C8—C9	108.03 (10)	F12—C10—F10	106.78 (12)
C7—C8—C10	109.71 (10)	H2A—C2—H2B	107.6
C8—O6—H6	109.5	H7A—C7—H7B	107.7
			
O1—C1—C2—C3	140.99 (13)	C4—C3—C5—F4	−63.00 (18)
O2—C1—C2—C3	−41.98 (19)	C4—C3—C5—F5	58.14 (18)
O3—C3—C4—F1	−58.76 (18)	C4—C3—C5—F6	176.95 (16)
O3—C3—C4—F2	−178.84 (14)	C5—C3—C4—F1	58.71 (18)
O3—C3—C4—F3	62.33 (18)	C5—C3—C4—F2	−61.37 (19)
O3—C3—C5—F4	52.05 (17)	C5—C3—C4—F3	179.80 (15)
O3—C3—C5—F5	173.18 (13)	C6—C7—C8—O6	50.77 (14)
O3—C3—C5—F6	−68.01 (19)	C6—C7—C8—C9	167.43 (11)
O6—C8—C9—F7	−179.87 (11)	C6—C7—C8—C10	−71.45 (13)
O6—C8—C9—F8	−57.98 (15)	C7—C8—C9—F7	57.53 (15)
O6—C8—C9—F9	61.31 (14)	C7—C8—C9—F8	179.43 (12)
O6—C8—C10—F10	−63.45 (14)	C7—C8—C9—F9	−61.29 (14)
O6—C8—C10—F11	55.60 (15)	C7—C8—C10—F10	62.26 (14)
O6—C8—C10—F12	178.20 (11)	C7—C8—C10—F11	−178.69 (12)
C1—C2—C3—O3	50.06 (17)	C7—C8—C10—F12	−56.09 (15)
C1—C2—C3—C5	−72.71 (15)	C8—C7—C6—O4	139.87 (11)
C1—C2—C3—C4	166.70 (12)	C8—C7—C6—O5	−42.93 (17)
C2—C3—C4—F1	178.96 (14)	C9—C8—C10—F10	−178.42 (11)
C2—C3—C4—F2	58.88 (18)	C9—C8—C10—F11	−59.37 (15)
C2—C3—C4—F3	−59.96 (18)	C9—C8—C10—F12	63.22 (15)
C2—C3—C5—F4	177.75 (13)	C10—C8—C9—F7	−62.79 (15)
C2—C3—C5—F5	−61.12 (17)	C10—C8—C9—F8	59.10 (16)
C2—C3—C5—F6	57.69 (19)	C10—C8—C9—F9	178.39 (11)

### Hydrogen-bond geometry (Å, °)

**Table:**

*D*—H···*A*	*D*—H	H···*A*	*D*···*A*	*D*—H···*A*
O1—H1···O5	0.84	1.83	2.6653 (14)	178
O3—H3···O2	0.84	2.01	2.7296 (14)	144
O3—H3···O1^i^	0.84	2.45	2.9561 (15)	120
O4—H4···O2	0.84	1.82	2.6635 (13)	178
O6—H6···O5	0.84	2.03	2.7502 (13)	144
O6—H6···O4^ii^	0.84	2.41	2.9276 (13)	121
C2—H2A···O3^ii^	0.99	2.36	3.2773 (16)	153
C7—H7A···O6^i^	0.99	2.32	3.2340 (14)	153

Symmetry codes: (i) *x*−1, *y*, *z*; (ii) *x*+1, *y*, *z*.
